# Perinatal care with a view to preventing cerebral palsy

**DOI:** 10.1111/dmcn.14754

**Published:** 2020-11-29

**Authors:** Nadia Badawi, Sarah Mcintyre, Rod W Hunt

**Affiliations:** ^1^ Grace Centre for Newborn Care Children's Hospital at Westmead Sydney Children's Hospital Network Sydney NSW Australia; ^2^ Cerebral Palsy Alliance Research Institute Specialty of Child & Adolescent Health Sydney Medical School Faculty of Medicine & Health The University of Sydney Sydney NSW Australia; ^3^ Department of Paediatrics Monash University Melbourne VIC Australia; ^4^ Neonatal Research Clinical Sciences Murdoch Children's Research Institute Melbourne VIC Australia; ^5^ Monash Newborn Monash Health Melbourne VIC Australia

## Abstract

Birth prevalence of cerebral palsy (CP) is declining in high‐income countries, to as low as 1.4 per 1000 live births in the most recent international reports. This represents a 35% reduction in birth prevalence over a 15‐year period. This reduction is underpinned by a heightened focus of attention towards all aspects of CP, including: increased awareness, better data collection, development of national networks and registries, an explosion of research in basic science, perinatal care, neonatal neurology, public health, early detection, and targeted early intervention. Quick uptake of evidence into practice has ensued and overall improvements in clinical care occurred concurrently. It is anticipated that with continued partnerships with families, ongoing research driving further clinical improvement and vice versa, birth prevalence and severity of CP will further decline and the focus will shift to prevention in low‐ and middle‐income countries.

**What this paper adds:**

- Research in the field of perinatal care and cerebral palsy (CP) prevention has increased significantly.
- In high‐income countries, increased awareness of CP and scientific advances have improved clinical care.
- Population‐based registers have limitations but remain the best mechanism to quantify birth prevalence of CP and accurately track trends.
- There have been recent reductions in the birth prevalence of CP.

## What this paper adds

- Research in the field of perinatal care and cerebral palsy (CP) prevention has increased significantly.
- In high‐income countries, increased awareness of CP and scientific advances have improved clinical care.
- Population‐based registers have limitations but remain the best mechanism to quantify birth prevalence of CP and accurately track trends.
- There have been recent reductions in the birth prevalence of CP.

**Abbreviation**
NICU: Neonatal intensive care unit

Cerebral palsy (CP) is a condition that essentially, but not exclusively, involves the motor system – originally thought to result from a static brain lesion resulting in a dynamic motor impairment that evolved and often deteriorated with advancing growth and development. Historically the condition had an associated sense of ‘fait accompli' that left clinicians focused on managing the complications of the condition. At that stage, we understood that brain injury was permanent; that the brain differed from other organs in the body because of its inability to regenerate and repair. The notion that the condition, rather than the symptoms, could be improved was beyond comprehension for many. However, the last two decades have seen exciting developments in the field of CP research that have largely been driven and recognized by data collected into population‐based registers. These include genome studies, preventive strategies such as antenatal magnesium sulphate, neuronal rescue strategies such as therapeutic hypothermia, and the increased uptake of early detection enabling rigorous early intervention research. Improved understanding of risk factors and clinical phenotype alongside advances in perinatal care have been the foundation for this dramatic increase in CP research, which is now reaping rewards.

CP can be classified into two broad groups based on timing of the injury responsible for causing CP: individuals whose brain injury occurred in the prenatal/perinatal period and those that acquire a brain injury postnatally. One of the most exciting developments has been the significant decrease in the rate of prenatal/perinatal CP that has been reported recently by CP registers. The decrease was seen first in Europe when birth prevalence declined from 1.9 in 1000 live births in 1980 to 1.7 in 1000 live births in 2003.^1^ During this period, bilateral spastic CP decreased for those with normal and moderately low birthweight. Norway and Western Sweden reported a decline, again in bilateral spastic CP,^2^, ^3^ and in Australia the rate declined from 2.2 in 1000 live births in the mid‐1990s to a low of 1.4 in 1000 live births in 2014, the most recent birth year reported.^4^

## Infants With Prenatal/Perinatal Brain Injury Born Preterm

Around 45% of people diagnosed with CP were born preterm (≤37wks gestational age). There has been a dramatic drop in the rate of CP among infants born extremely preterm (≤27wks gestational age),^5^ along with an increase in survival without disability.^6^ The reasons for this are complex and multifactorial, but include antenatal strategies such as the implantation of one embryo with in vitro fertilization,^7^ the implementation of magnesium sulphate for neuroprotection,^8^ and corticosteroids administered to mothers antepartum for accelerating lung maturation^9^ in anticipated preterm birth. There is also high‐level evidence emerging for delayed cord clamping and caffeine for apnoea of prematurity – both of which could impact on causal pathways for infants born preterm.

Greater experience with infants born preterm has enabled staff to provide better care over time with improved resuscitation, less intubation, and increased use of nasal continuous positive airway pressure around the time of birth.^10^ There are now a number of reports throughout the world showing a significant reduction in severe intraventricular haemorrhage – particularly from 2010 onwards.^11^, ^12^, ^13^ We wait to see if the next CP register reports reflect a drop in hemiplegia in infants born preterm. With survival rates of all infants admitted to neonatal intensive care units (NICUs) increasing dramatically,^14^ better focus to improve neurodevelopmental outcomes for NICU graduates has driven the development of standardized NICU protocols.^15^ Other data monitoring initiatives, such as the Australian and New Zealand Neonatal Network and the Canadian Neonatal Network, compare survival and other outcomes between NICUs allowing for benchmarking between units and assessment of the impact of quality improvement initiatives at a national level.^16^

## Infants With Prenatal/Perinatal Brain Injury Born at or Near Term

In the most recent international report, the Australian Cerebral Palsy Register identified a considerable drop in the rate of CP among term born infants who account for over half of the infants with CP.^4^ We wait in anticipation for the Surveillance of Cerebral Palsy Europe to report more recent birth years to see if this finding is replicated in Europe. Developments in antenatal ultrasonography have allowed more accurate diagnosis of congenital anomalies thereby ensuring that infants with major congenital anomalies are born at large maternity hospitals with co‐located surgical centres and appropriate obstetric, neonatal, and surgical expertise. This avoids the hazards of a late diagnosis which include clinical deterioration and neurodevelopmental compromise before postnatal transfer to a surgical centre. The improvement in antenatal ultrasonography and diagnosis^17^ has had a real impact on practice. In the past an infant with undiagnosed cardiac disease would become cyanosed, acidotic with low blood pressure, and possibly suffer ischaemic brain injury before transfer. This has become extremely rare in Australia. It is also possible that some families who have an infant with an early antenatal diagnosis associated with a brain anomaly detected on fetal imaging may elect to have the pregnancy terminated.^18^ Tandem mass spectrometry has also allowed earlier diagnosis of an expanded group of inborn errors of metabolism (some of which may predispose infants to cerebral insults) before clinical deterioration, avoiding cerebral metabolic injury.^19^

A proportion of term born infants who experience intrapartum hypoxia have benefitted from therapeutic hypothermia which has become the standard of care in NICUs in high‐income countries.^20^ Hypoxic ischaemic encephalopathy cannot be predicted antenatally in many cases,^21^, ^22^ however with assistance from neonatal emergency transfer services, passive and active cooling is commenced earlier, ensuring instigation of therapeutic hypothermia within 6 hours of birth for the great majority of these infants.

## Infants With Postnatally Acquired Brain Injury

Rates of postnatally acquired CP have also decreased.^4^ Factors driving this decrease include screening during pregnancy for Group B streptococcus with administration of intrapartum antibiotics, vaccinations avoiding meningitis, and improved perioperative care for congenital cardiac anomalies. Data collected by Departments of Health monitoring trends in infant and paediatric mortality, have stimulated the development of a number of public health policies including compulsory car seats, swimming pool fences to reduce the incidence of near drowning, and public awareness campaigns such as ‘Don't shake your baby'.

While these results are heartening, history tells us that we must avoid being complacent about both our current practice and these results. Recently in Australia there were reports of increased numbers of infants with kernicterus related to delayed detection of hyperbilirubinaemia. Infants from diverse cultural backgrounds seemed most at risk. This observation followed widespread implementation of policies recommending early discharge home from maternity hospitals.^23^ Recognition of this association challenges us to continue to pursue best practice and to avoid the risks that societal vulnerability inevitably brings. Population‐based CP registers are an excellent measure of the impact of new interventions and modes of care, both for bad and good, and *must* be maintained. Ongoing scrutiny of this data will also stimulate the next waves of research to further reduce the incidence and impact of CP, and stay on top of any unexpected increases.

## What About the Future?

### Reducing the impact of preterm birth

With preterm birth associated with 45% of cases of CP, it follows that reducing the rate of preterm birth will likely decrease the rate of CP further. A recent Cochrane Review recommends four key interventions to reduce preterm birth: midwife‐led continuity models of care, screening for lower genital tract infections, zinc supplementation for pregnant females without systemic illness, and cervical cerclage for females with a singleton at high risk of preterm birth.^24^ Other promising interventions include cervical pessary, cervical length assessment, and vaginal progesterone.

The Western Australian Preterm Birth Initiative has reduced preterm birth by 8% using a combination of health professional advice, public health and social media campaigns, and a preterm birth prevention clinic.^25^ It is now being expanded across Australia through the Australian Preterm Prevention Alliance, and will soon be replicated in Canada and the USA. Additionally, a ‘Birthing in our Community' programme for Aboriginal and Torres Strait Islander females has reduced preterm birth from 11.6% to 6.9% (a 40% reduction from the Indigenous baseline rate). This is a result of changes to pregnancy care designed to empower Indigenous females and families including increasing the Indigenous workforce, continuity of midwifery care, and integration of family support within a community hub.^26^ It is imperative that programmes such as this are fully funded for implementation (outside of the research setting) and adaptation to the local context throughout Australia.^27^

The immediate perinatal period is a time of extreme vulnerability for infants born preterm. There is an increasing amount of research interrogating optimization of resuscitation and ventilation. A recent study by Tracy et al. revealed that resuscitation equipment often does not meet the desired standards^28^ and adds to data from Polglase et al.^29^ which demonstrates that the inflammatory cascade that is unleashed in the delivery suite can adversely impact the brain. Multiple other studies have shown an association between ongoing mechanical ventilation and subsequent neurological impairment.^30^ This will continue to be an area of fruitful research.

Once admitted to the NICU, newborn infants are highly dependent on technology and engineering skills. There is increasing recognition of neuromonitoring alongside cardio‐respiratory monitoring in the NICU. Amplitude integrated electroencephalogram is now commonplace in NICUs in high‐income countries. Newer neuromonitoring modalities such as near infra‐red spectroscopy provide insights into cerebral oxygen saturation, and are currently being interrogated for their ability to predict outcome, and perhaps aid in improving survival and neurodevelopmental outcomes. Vesoulis et al.^31^ demonstrated using near infra‐red spectroscopy that there was a significant difference in the mean cerebral oxygen levels between infants who went on to develop severe intracranial haemorrhage, and those who did not.

Beyond monitoring, specific neuroprotective strategies such as erythropoietin have shown early promise^32^ and we await long‐term results of its effect on neonatal stroke.^33^ A meta‐analysis of studies of erythropoietin administered to infants born preterm as a neuroprotective strategy showed a trend towards lowering the combined outcomes of any neurodevelopmental impairment. However, a larger definitive randomized controlled trial demonstrated that prophylactic high dose erythropoietin given to infants born extremely preterm did not appear to be neuroprotective at the dose investigated.^34^

### For the term born infant at risk of CP

Antenatal factors such as intrauterine growth restriction, congenital anomalies, and infections are some of the biggest risk factors for term newborn encephalopathy^21^ as well as CP.^35^ These risk factors often occur together, and causal pathways are more often responsible for aetiology rather than one single cause. Drugs to reduce intrauterine growth restriction in animals (e.g. creatine) have already been investigated in preclinical models^36^ and human trials have commenced in mothers of infants with intrauterine growth restriction to investigate the possible benefit of melatonin.^37^ To obtain further reductions in an already low rate of CP, we have to focus attention on rarer preventable risk factors. Cytomegalovirus is an uncommon but significant and potentially preventable cause of antenatal cerebral injury and CP.^38^, ^39^ A recent international consensus guideline for the prevention of cytomegalovirus in pregnancy and treatment of congenital cytomegalovirus noted that education and public health prevention strategies for pregnant females were beneficial – a number of these are now occurring around the world. This paper also called for a debate for universal neonatal screening for cytomegalovirus for early detection and intervention.^39^

For the small proportion of term born infants who suffer a lack of oxygen or cerebral blood flow around the time of birth, therapeutic hypothermia is an important neuro‐rescue therapy reducing both mortality and morbidity amongst survivors of birth asphyxia.^40^ However, apoptosis and neuroinflammation are not entirely prevented with therapeutic hypothermia, and there will be a place for other neuroprotective strategies to be used in combination with therapeutic hypothermia. Some of these will have different mechanisms of action and may even repair brain tissue.^41^ Currently, the most promising are Phase III multicentre randomized controlled trials in the USA and Australia/New Zealand comparing therapeutic hypothermia alone to therapeutic hypothermia plus erythropoietin in term born infants with hypoxic ischaemic encephalopathy,^42^ and results should be available from these trials over the next few years. Other combination strategies currently in Phase I and II trials include therapeutic hypothermia + melatonin, allopurinol, xenon, and magnesium sulphate, and preclinical data is also promising for mesenchymal stem cells, N‐acetyl cysteine, and cannabinoids. The emerging field of precision medicine may help us elucidate the best treatment for each infant.

### For infants in the NICU or recently discharged

There is emerging evidence that targeted early intervention including cognitive input may also improve neurodevelopmental outcomes.^43^ Fortunately, tools such as the General Movements Assessment and increasing use of neuroimaging modalities like magnetic resonance imaging mean that we can now reliably identify infants at high risk of CP as young as 3 months of age.^44^ There is still much to be learnt about the most effective early intervention strategies for those infants at high risk of CP, and their early recognition provides opportunities for families to participate in this important research. However, knowledge, improved neuroimaging, and sensitive clinical assessment is not enough. For trials of early intervention to be completed in a timely way, the entire neonatal sector, beyond the dedicated clinician scientists who engage in research in this field, needs to tackle the issue as part of provision of routine newborn intensive care. To fulfil this brief, we must fully understand the established risk factors for CP and be prepared to have authentic conversations with parents of infants at high risk of CP whilst in the NICU, or soon after discharge. Recognition of the problem will minimize parental stress and improve child and family outcomes, rather than the historically held belief that parents must be protected from bad news and be allowed to enjoy happy days of ignorance while they last.

### Tackling the ‘unpreventable'

Congenital anomalies are found in nearly 25% of people with CP and this group have largely been excluded from research.^45^ In addition, children with CP and congenital anomalies are likely to have a more severe phenotype. There is a large collaboration underway between the Australian Cerebral Palsy Register, the Surveillance of Cerebral Palsy Europe, and EUROCAT analysing data on over 8000 children with CP. Congenital anomalies are common among those with postnatally acquired CP as well as prenatal and perinatal CP. This is important as the primary prevention of congenital anomalies antenatally may also prevent CP in some cases. There is likely to be a large overlap with genomics (in particular with this group), and recognition of this association has led to the development of the International CP Genomics Consortium (www.icpgc.org). Large numbers of DNA and clinical information from affected infants, children, and their parents will be necessary to better understand these complex inter‐relationships, and a database is currently being built (The CP Commons) which will mean genomic and clinical data can be shared across the world, to maximize efficiency and outcomes. One other area that demands more investigation is perinatal stroke in term born infants. Rates of hemiplegia have not declined in the registers,^46^ and although this is a condition amenable to improvement with early intervention, prevention remains elusive.^47^

We should also embrace the technologies and strategies that our engineering and computer science colleagues are beginning to provide. The use of artificial intelligence has potential to further improve our diagnostic capacity and management of conditions that are currently susceptible to the variation in human opinion. Machine learning to predict fetal health status^48^ and preterm birth^49^ is already advancing.

### Globally

We have presented a very focused high‐income country view describing rates of CP and advances in management particularly where CP registers exist.^50^ Future research should include socio‐economic inequity in high‐income countries. However, globally there are also advances being measured by differing mechanisms of data collection, all of which have a margin of error.^51^ There are a number of CP registers being developed in regions of low‐ and middle‐income countries such as Bangladesh, Sri Lanka, Nepal, Indonesia, Ghana, and Vietnam; all of which are providing essential information and services for children in these areas. Early findings are showing these countries have higher rates of CP with greater severity, lower life expectancy, and strong associations with social risk such as extreme poverty.^52^ This was also seen in a recent cross‐sectional prevalence study in rural Uganda.^53^ There are preventable causes of CP that differ across these regions. Many of these countries are experiencing increasing affluence, especially in the cities, and are likely to be building NICUs. These countries may benefit from the experience of high‐income countries if they are to avoid the increase in CP seen in our NICUs in the 1970s and 1980s.^54^ This has potential to have a huge impact since at least 50% of all infants subsequently diagnosed with CP in high‐income countries spent time in either a NICU or a special care unit.

### Consumer involvement is crucial

For research investigating prevention and reduction of severity of CP to proceed as quickly as possible, we must ensure that families and individuals with CP are integrally involved in research development and conduct, and the translation of findings into clinical practice. Consumer generated priorities have driven a great deal of the research that has occurred over the last 10 years. One of the most important priorities for family members and people with CP is the prevention of CP.^55^ A member of our research consumer group ‘CP Quest' was recently involved in the translation of the Australian Cerebral Palsy Register Report. She stated: ‘I can tell you as someone living with cerebral palsy, that those figures [rates of CP decreasing by one‐third] and the possibilities they suggest for the future bring a huge smile to my face. Health professionals and researchers have greatly reduced the possibility of young kids having to grow up and go through what I and so many others have'.

## Conclusion

We describe a decline in the birth prevalence of CP that results from a number of different strategies and interventions. In the first instance, better knowledge about the true birth prevalence and trends of CP, through the development of population‐based registers, has promoted and focused research in a way that has driven interrogation of preventive strategies, improvements in clinical care from conception into childhood, earlier detection of CP, and implementation of early intervention strategies. With ongoing endeavours in this area, we anticipate further reductions in the birth prevalence of CP and continued improvements in quality of life for those living with CP.

## Data Availability

Data sharing is not applicable to this article as no new data were created or analyzed in this study.
